# Data science's cultural construction: qualitative ideas for quantitative work

**DOI:** 10.3389/fdata.2024.1287442

**Published:** 2024-08-14

**Authors:** Philipp Brandt

**Affiliations:** Department of Sociology, Sciences Po/CSO, Paris, France

**Keywords:** data science, emergence, expertise, professions, reflexivity, computational social science, social network analysis, computational ethnography

## Abstract

**Introduction:**

“Data scientists” quickly became ubiquitous, often infamously so, but they have struggled with the ambiguity of their novel role. This article studies data science's collective definition on Twitter.

**Methods:**

The analysis responds to the challenges of studying an emergent case with unclear boundaries and substance through a cultural perspective and complementary datasets ranging from 1,025 to 752,815 tweets. It brings together relations between accounts that tweeted about data science, the hashtags they used, indicating purposes, and the topics they discussed.

**Results:**

The first results reproduce familiar commercial and technical motives. Additional results reveal concerns with new practical and ethical standards as a distinctive motive for constructing data science.

**Discussion:**

The article provides a sensibility for local meaning in usually abstract datasets and a heuristic for navigating increasingly abundant datasets toward surprising insights. For data scientists, it offers a guide for positioning themselves vis-à-vis others to navigate their professional future.

## 1 Introduction

Digital transformation has impacted many areas of social life, including politics (Schradie, 2019; Bail, 2021), news (Christin, 2020), and the economy (Zuboff, 2019), particularly through social media. The impacts differ, ranging from efficiency gains to polarization and misinformation, but they have in common the entanglement of the novel “data scientists” profession in these changes. This new role has remained obscure despite its salience and older foundations (González-Bailón, 2017). While the ambiguity has likely had benefits for data science (Dorschel and Brandt, 2021), data scientists have struggled with the lack of clarity (Avnoon, 2021). This article asks how the emerging data scientist community has defined their novel role on social media and addresses methodological issues that come with studying an emergent case.

The problem is complicated as strategies of established professions are not immediately available to an emerging profession. Evidence shows how existing professions respond to the ongoing changes in organizational settings (see, e.g., Greenwood et al., 2002; Armour and Sako, 2020; Goto, 2021), but traces of data science's self-definition first appeared on the Internet in blog posts, or on Twitter. A now-classic tweet serves as an example and a working definition: “Data scientist (n.): Person who is better at statistics than any software engineer and better at software engineering than any statistician.”^1^ The definition presents data science as an expert role and, read verbatim, gives a sense of the quantitative and coding skills this work entails, but it does not try to be comprehensive or entirely clear and demands that any systematic analysis reconciles local specificity and the phenomenon's global salience.

The immediate questions of how much software engineering a statistician has to know or which parts have been answered by various training programs and textbooks (Schutt and O'Neil, 2013; Salganik, 2018; Saner, 2019; Dorschel and Brandt, 2021). A more puzzling question remains in the definition's imitation of a dictionary definition on social media, where that formalism was unnecessary and long before one existed in print. The style instead leveraged the lay view of expert work as jurisdictions of formal professions (Freidson, 2001). It connects the problem of data science's construction to discussions in the literature on expert knowledge and work. This literature has long developed a nuanced understanding of professions as a system of competitors (Abbott, 1988), emergent relational arrangements (Eyal, 2013), and their organizational dimensions (Muzio and Kirkpatrick, 2011). In contrast, the definition's playfully premature formalism highlights cultural processes underpinning emergent professions.

Culture has an everyday meaning and a technical meaning. Data scientists have recognized the role of culture in the everyday sense, at least sporadically and casually, in terms of “two cultures” in quantitative thinking (Breiman, 2001) or the “culture of big data” (Barlow, 2013). They mean characteristics of their work that do not follow purely technical or formal steps. Sociological theories of expert work acknowledge cultural processes in a more technical sense but often assign them less weight compared to other mechanisms, competition, informal relations, and organizational dynamics. Culture featured in Abbott's (1988) classic account in the background of the main argument as the “diagnosis, treatment, and inference” that jointly form the “cultural machinery of jurisdiction” (Abbott, 1988, p. 60). Culture also played an external role such as when public opinion creates problem areas that professions can claim as their jurisdictions (Abbott, 1988, ch.7). Fourcade's (2009) comprehensive analysis of economists and their history worked out this side in the interplay of economic culture and institutions, indicating that contexts shape economic theories, which, in turn, shape their environments.

Capturing meaning-making presents a unique challenge in an emergent setting where technological and economic forces converge with the ideas of professional pioneers. Cultural processes have shaped quantitative expertise for a long time (Porter, 1986, 1995; Desrosières, 1998), and data scientists have made a new iteration visible through their appearances in public discourse and popular culture.^2^ Several studies have demonstrated the complexity of this outside relationship between experts, and their publics (e.g., Wynne, 1992; Epstein, 1996), which may in part stem from mismatching views as outsiders have low regard for the technically advanced knowledge that experts value (Abbott, 1981). This article addresses its motivating question of how the data scientist community has defined their role from a cultural perspective that builds on Burke's (1945) notion of *A Grammar of Motives*. This modern interpretation, which John Mohr introduced as “computational hermeneutics” (Mohr et al., 2013), extends research on expert work into the digital age and gives the intuition data scientists have had since their beginning a rigorous foundation.

The analysis integrates recent arguments for understanding culture in professions into novel computational procedures for formal measures of culture. Spillman and Brophy (2018, p. 156) stressed the “implicit and explicit claims about the practical or craft knowledge” in addition to the common focus on abstract or technical expertise. Whereas, they illustrated their argument with reference to documentary and ethnographic analyses, this study moves to the digital context, where data scientists often discussed their role. It uses a large dataset of tweets to capture public discussions and draws on advances among scholars of culture around using computational social science techniques (see Edelmann et al., 2020). The focus in qualitative research on “vocabularies of motive about work” (Spillman and Brophy, 2018, p. 159) links to methodological ideas for recovering cultural features from large numbers of textual documents to reconstruct the meaning that actors assign to situations (Mohr et al., 2013, 2015).

This conceptual approach guides a computational analysis of data science's cultural construction. The combination informs an analytical strategy for studying expert work, meaning construction, and disputes on social media where they unfold in public. It is able to track meaning-making on different levels to capture data science's local definition and global salience. The results reveal data science within the larger changes of the digital era as a rhetorical strategy for circumventing established groups, their leaders, and legacies to adapt old skills to contemporary issues (see Frickel and Gross, 2005; Suddaby and Greenwood, 2005). They show an arrangement of actors and themes that suggests new ethical and technical ideas and practical challenges around implementing them as a previously unreported motive of data science's construction. To develop this argument, the article first introduces the data science case, the reflexive analytical approach, and the empirical strategy before summarizing and discussing the observations.

## 2 Data science as an emergent profession

Data scientists have told origin stories that centered on Facebook and LinkedIn in their early startup days, struggling to get users to connect and navigate the then-new world of social media (Hammerbacher, 2009; Davenport and Patil, 2012), but the data science label first appeared in academic circles during the 1990s and early 2000s (e.g., Hayashi, 1998; Cleveland, 2001), and many underlying ideas are much older (Donoho, 2015; González-Bailón, 2017). Data scientists recognize their ties to established quantitative expertise and present their integration of it with computer sciences as a distinguishing feature (e.g., Schutt and O'Neil, 2013).

Such origin stories and programmatic definitions do not necessarily spread along direct and linear paths. Historical research of quantitative work and thinking has shown how quantitative experts shared technical ideas about their work in ways that indicate cultural processes (Porter, 1995), such as through “evidential cultures” of data analysis (Collins, 1998). Following the practical work in social media startups during the mid to late 2000s, data science has spread into various industries and public services, all the way to the Obama administration (Hammerbacher, 2009; Davenport and Patil, 2012; Lohr, 2015; Smith, 2015). Its appearance and diffusion indicate another iteration in the long and storied history of quantitative expertise as it extends into the digital age.

Sociological accounts of data scientists have studied data science from different perspectives, beginning with their emergence (Brandt, 2016). Some research shows that data scientists struggle with integrating the multiple competencies and areas of expertise of their roles in their workplaces (Avnoon, 2021). Other research suggests that precisely the ambiguities that undergird the data science role, at least on the level of the larger educational and economic fields, have advanced data science's professional recognition (Börner et al., 2018; Dorschel and Brandt, 2021). Journalistic accounts of data science described socio-technical arrangements (e.g., Lohr, 2015), where the sociology of expertise would partly locate data science's roots (Eyal, 2013). Social scientists have even reflected on their own relationship with data science, both conceptually, in STS (Ribes, 2019), and practically, in quantitative research (González-Bailón, 2017; Salganik, 2018), and stressed the threats to society (O'Neil, 2016; Eubanks, 2018). These critical perspectives have initiated concerns with ethics among data scientists (Loukides et al., 2018), another familiar step in the development of professions (Abbott, 1983). The question of how data scientists resolve the ambiguity of their new role as a group a cultural process has remained unexplored.

## 3 Empirical strategy

### 3.1 A reflexive perspective

The early discussions of data science on social media offer a promising opportunity for shedding further light on this new case, but an analysis of data science's cultural construction on social media faces challenges as some who contribute to it may not self-identify as data scientists, and new ideas may not immediately appear relevant. For example, some social scientists helped define data science without affiliating with the new group (e.g., González-Bailón, 2017; Salganik, 2018). This problem raises questions about the analyst's perspective, which anthropologists and sociologists discuss as reflexivity (Gouldner, 1970; Geertz, 1988). Reflexivity has gained new attention and motivated the idea of “asymmetric comparisons,” wherein an analysis captures “the larger diversity in the world” (Krause, 2021, p. 9). These comparisons address the problems with an analysis of data science on social media by suggesting comparisons between narrower views of data science to broader observations that are missing initially.

Quantitative research often aims for representative samples and conceives of foregone observations as a problem of missing data that introduces biases. It has addressed that issue systematically for a long time (e.g., Kim and Curry, 1977; Little and Rubin, 2019). Assuming that all relevant variables are available, which quantitative methodologists acknowledge is not always the case, the main distinction is between missing information on single items for respondents and entire units that did not respond (Loosveldt and Billiet, 2002; Peytchev, 2013). The debate further discusses missing data in specific areas of research, such as social networks, which raise questions about the completeness of the units used for studying them (e.g., Kossinets, 2006).

Both perspectives can help shed light on data science's formation. For an asymmetric data science comparison that the qualitative perspective counsels, the quantitative perspective would mean adding information on a set of data scientists for which some information may be missing. Such a case should consist of a larger network boundary to reveal the implication of the initial boundary decision. Finally, it seems unlikely that research subjects routinely discuss relevant social dynamics directly (Jerolmack and Khan, 2014), especially as they still define their identity, such as data scientists. The boundary (Laumann et al., 1983) needs to capture more and less overtly related types of content. This complication captures a specific challenge in the larger program of bringing qualitative ideas to quantitative research (e.g., Mützel, 2015; Evans and Foster, 2019; Brandt, 2023).

### 3.2 Observations and operationalization

This cultural analysis of data science's emergence on social media is part of a larger project that began with field observations of the early data science community in New York City between 2012 and 2015. Those observations covered public events where data scientists presented their work and views of the field. They captured data scientists from close proximity in an important setting but missed many other settings, as well as data science's ongoing construction after the fieldwork ended. This article analyzes the subsequent discussions of data science issues on Twitter, avoiding some constraints from in-person observations even as new limitations come up, which I discuss below. Twitter was ubiquitous in the community during the field observations, where data scientists often mentioned their Twitter accounts when they introduced themselves to audiences. I started following data scientists whom I encountered and added others that appeared in my timeline and seemed relevant. I avoided a general search to ensure consistency with the field observations that had identified central perspectives in the larger data science discussion.

The analysis follows Mohr et al. (2013) to reveal data science's cultural construction on Twitter as a “grammar of motives” that considers “what was done (act), when or where it was done (scene), who did it (agent), how [they] did it (agency [that is, by what means]), and why (purpose)” (Burke, 1945, p. xv). Mohr et al. (2013) proposed formal methods for extracting motives from quantitative data. On Twitter, the data scientists (and other users) are “actors,” and Twitter is the “agency” that allows individuals, organizations, and other groups to register, publish tweets of 280 characters or less, follow other accounts to see their tweets, and react to those tweets via liking them or responding. These activities were the “acts.” Both the acts and Twitter, as infrastructure, remained largely stable throughout this analysis and did, therefore, not contribute to an analysis of data science's ongoing construction.^3^

Purposes and scenes are the relevant analytic dimensions in addition to the actors. The analysis identifies purposes through Twitter's hashtag functionality. Twitter allows users to include hashtags (#) followed by 1 grams, such as #ArabSpring, #MeToo, or #datascience. These hashtags highlight causes that a tweet seeks to promote and link to other tweets with the same hashtag. I use weighted log odds ratios to identify dominant purposes. For revealing “scenes,” Mohr et al. (2013) used text analytic methods, which I apply to tweet texts. Table 1 summarizes these connections between concepts, operationalization, and analytic techniques (columns 1, 2, and 7). The respective sections provide details on each technique. Together, they reveal key dimensions of data science's cultural construction on social media.

**Table 1:** Sample design and raw data structure for asymmetrical comparison.

**Concepts**	**Operationalization**	**Small data**	**Large data**			**Analytic techniques**
			**Total**	**First degree**	**Second degree**	
Actors	Users	395	455,344	136	246	SNA
Purposes	Hashtags	475	335,337	148,718	186,607	Weighted log odds ratios
Scenes	Tweet texts	1,025	752,815	294,646	464,137	LDA

SNA, social network analysis; LDA, latent Dirichlet allocation.

### 3.3 Data structure

Twitter's digital infrastructure offers access to vast observations. Concepts from the sociology of professions and expertise, outlined in the introduction, guided the original collection of relevant tweets, but the digital transformation has made vast observations of social activities easily accessible. To design an asymmetric comparison for a reflexive analysis (Krause, 2021), I used Twitter's API to obtain the publicly available timelines of the accounts that posted the tweets in the initial dataset, the connections between accounts, and accounts missing from the initial dataset. The design responds to methodological concerns with capturing actors and what they have to say.

I introduce an intermediary comparison for better understanding the effect of changing boundary conditions and specifying data science's emergent contours. When developing his hermeneutic perspective, Burke (1945, p. xix–xx) noted that “an agent might have [their] act modified (hence partly motivated) by friends (co-agents) or enemies (counter-agents).” In this reflexive analysis, my Twitter “friends”—Twitter-speak and Burke's conceptual language overlap for what network analysts call first-degree neighbors—may have captured a more focused discussion.^4^ The idea of a counter-agent makes sense for the accounts that my ongoing observations missed in as far as they possibly covered a broader discussion. Social network analysis language refers to these accounts as second-degree neighbors. The subsequent analysis captures the “larger diversity in the world” (Krause, 2021) by comparing (1) the patterns that emerge from the dataset of actively collected tweets to those of digitally obtained full timelines and, within those timelines, (2) patterns in friends tweets to those in strangers tweets, or first and second-degree neighbors.

The initial dataset consisted of the tweets that I collected from my timeline as insightful moments from the project's theoretical perspective, beginning in March 2017. This analysis includes tweets until March 2020, when the coronavirus pandemic took over much of the data science conversation. During this time, I manually collected 1,025 tweets from 395 accounts (Table 1, column 3). The next section summarizes their content. These observations missed the vast majority of tweets these users posted and shared. I obtained additional tweets by these users and their relations through the Twitter API (Table 1, columns 4–6). The resulting dataset includes 455,344 second-degree Twitter ties and a corpus of 752,815 tweets that explicitly indicated English as their language.^5^

### 3.4 Data science on Twitter

This section summarizes data science-related tweets as a first illustration of how Twitter featured in data science's definition, capturing talk of positions, expertise, promises, and threats. Several tweets in the small dataset discussed jobs, which are critical for claiming an area of work (Abbott, 1988). One tweet from November 2018 mentioned an opening in Facebook's Core Data Science team. Others advertised an opening at Detroit's Innovation Team to data scientists who look in that region, or a vacancy at MindGeek, which that tweet identified as the owner of an adult content website.^6^ Many others commented on hiring issues, warning, for example, of a lack of demand or that those hiring data scientists mainly look for versions of themselves. Some were quite reflective, noting, for example, that “In my experience, people who [do] data science well tend to get PhDs, but the PhD itself is negative preparation for the job.” In a topic as straightforward as work, tweets can capture more nuance than the popular celebrations or critiques of their large demand capture.

Data science also involves technical expertise, which seems much harder to fit into tweets. Some tweets have taken a light take on methods, joking, for example, how someone may falsely underestimate their significance for data science or, conversely, that some use the common perception of methods as leading to rigor without understanding them. Others share more profound thoughts. Yann LeCun, a pioneer in artificial intelligence and the first director of NYU's data science institute, used the idea of methods across data work, painting, or musical composition to explain the meaning of deep learning.^7^ As for the job tweets, these tweets develop technical data expertise instead of broadcasting simple lists of skills.

Many tweets that mentioned data science did not shed additional light on data science's professional construction. I recorded some of them, such as one in which Kirk Borne, a data science popularizer, announced a webinar and used many hashtags, presumably to increase its visibility. This tweet, and a few like it, entered the observations as a record of promotions that mentioned data science without developing its meaning.

The tweets so far illustrate how the data science community discussed the meaning of jobs or methods and their promise online. Other tweets problematized the question of the community itself. The idea of ethics in data science flared up occasionally, and prominently so in the fall of 2018 when well-known data scientists Hilary Mason and DJ Patil published a book titled *Ethics and Data Science* together with Mike Loukides (Loukides et al., 2018). Another instance of community formation unfolded as a collective reaction to bullying when several data scientists spoke up against one account formally affiliated with data science for having bullied a member of their community. While these examples capture clear moments of community building, others remain more subtle.

This summary shows that Twitter served, at least in some instances, as a discursive space for defining data science. The subsequent analysis models the community's collective construction of data science on Twitter in terms of its underlying motives and across varying boundary specifications.

## 4 Analysis and results

### 4.1 Actors

The first analytical step considers actors, the Twitter accounts that posted tweets about data science. Burke (1945, p. xix–xx) suggested that agents “subdivide” into groups. This step first analyzes the group structure of the 395 accounts that constitute the small dataset of qualitative observations with respect to the connections between them as well as connections in the large dataset of 455,344 accounts they followed. The “walktrap” community finding algorithm, a standard function in R's igraph package (Csardi and Nepusz, 2006) that builds on the widely used modularity measure (Pons and Latapy, 2005) with a focus on communication settings (Smith et al., 2020), revealed the relational subdivisions of these actors. It uses random walks to partition a network into groups of nodes with dense connections between each other and sparse connections to other nodes.

I begin with the most comprehensive dataset. The large dataset includes 455,344 accounts, all contacts followed by the 395 accounts from the qualitative observations. I created a bipartite network of these following relations, with the 395 focal accounts on one level and the ones they follow as the second level. I projected this bipartite network on the level of the focal nodes, retaining ties between nodes that follow the same other account, weighted by the number of common accounts, and applied the community finding algorithm. This strategy ensures the interpretability of the structural characteristics in terms of the focal nodes while considering a wider structural context. Substantively, it captures that although two accounts may not follow each other, say, two junior data scientists where one is in a university and another in a startup, they may still follow the same prominent accounts. The weighting accounts for the number of accounts in which the two data scientists may share an interest.

The algorithm identified two main communities and a third, smaller community. This result amid an average out-degree of over one thousand nodes for the focal accounts before the projection indicates a strong interest in other Twitter accounts. The two larger groups consist of 265 and 101 accounts and the smaller one of 26 accounts. The modularity score is 0.08, indicating substantial integration. Only 14% of the node pairs have no accounts in common among those they follow, while 49% share ten or more. Qualitative inspection revealed that the largest one consists of more hands-on accounts, including software coders in applied roles but also academics from different disciplines and a few commentators from media and industry, but these two groups of accounts more distinctively cluster in the second larger group, which includes less of the hands-on accounts, capturing the role of often self-described “thought leaders” in these early data science discussions. This structure offers a plausible image of data science's emergent community structure that includes core contributors and some hangers-on. While it reflects abundant records, it is simple and does not indicate any underlying motives.

**Figure 1:**
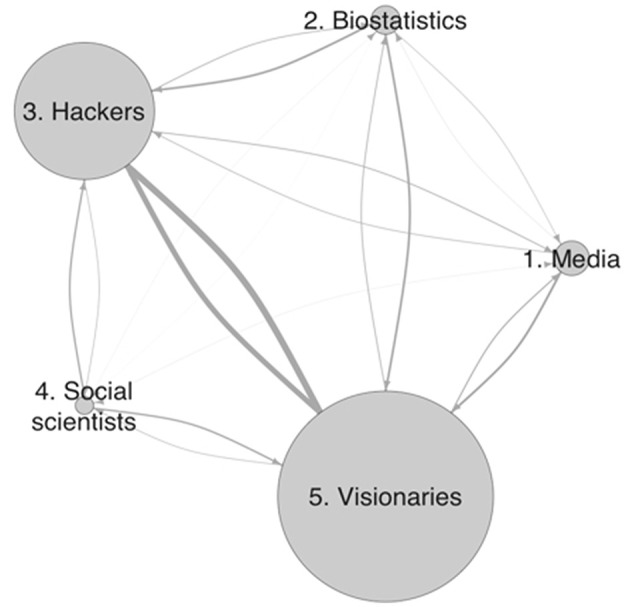
Network of groups and their aggregate relations.

The next analytical step changes perspective. It considers the immediate relational structure within the tighter boundary of the small dataset of 395 accounts and the 11,580 ties between them.^8^ The community detection produced five groups with a modularity score of 0.15.^9^ Figure 1 shows this network on an aggregate level where the node sizes indicate the number of accounts in each group (reported in separate discussions below); the arrows between them bundle individual ties from one group to another. The line thickness of the arrows indicates the followership ties from the sender-group perspective. Each group has at least one connection to each other group, except for the media group, where no account follows any account in the social scientists group. On the aggregate level, the strong connections stand out between what I will be introducing as the hacker group and the visionaries, with 123 and 104 ties in the respective directions. Both groups are large and have intuitive links to data science's emergence, but while their interconnection is strong, they are much weaker than the internal connections, consisting of 1,919 and 4,560 ties, which led to the clusters that I discuss next. This network of only direct following relations recovers existing groups that contributed to early data science conversations on Twitter.

**Figure 2:**
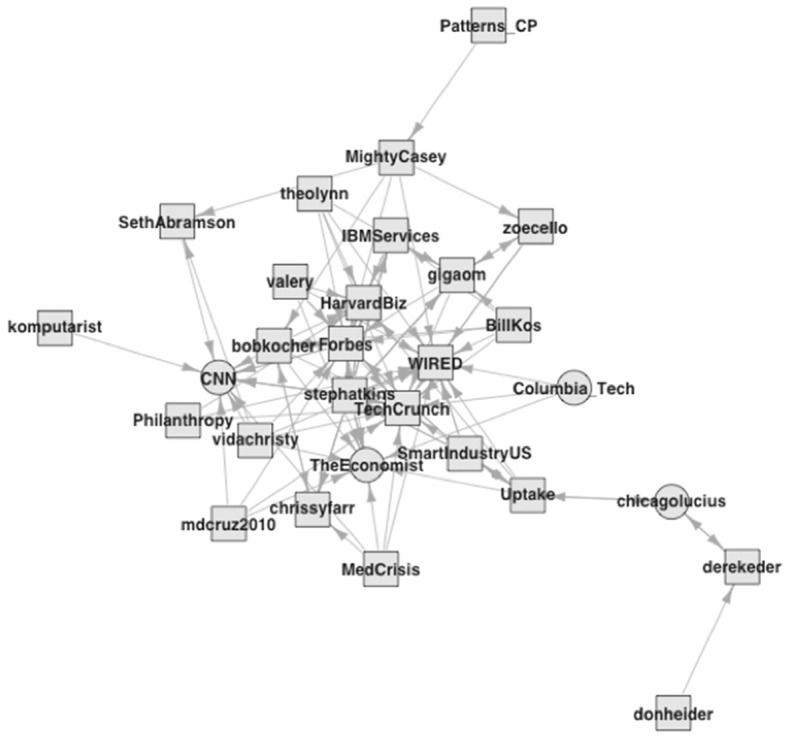
Followership relations within the media group (1).

The first group contains prominent accounts (Figure 2; squares represent second-degree accounts from the data collection perspective, and circles represent first-degree accounts). The 29 accounts in this group have a dense core but otherwise moderate interconnections with a density of 0.14.^10^ Several belong to newspapers and magazines, such as Forbes, The Economist, CNN, WIRED, and TechCrunch, an online publisher covering the tech industry. These accounts capture data science's cultural context (Abbott, 1988; Fourcade, 2009), signaling the broader interest in data issues during data science's emergence. There are also HarvardBiz and Columbia_Tech, two university-affiliated accounts, and IBM Services from the technology industry, which all represent official and corporate actors. Circular node shapes indicate first-degree accounts, which capture one of Burke's ideas on actors. This group includes only a few direct neighbors, such as CNN, The Economist, and chicagolucius, a personal account of a user who indicates roles as a chief data scientist and data officer with the City of Chicago.^11^ The outsized salience of second-degree accounts here increases exposure to their tweets through retweets. This group reflects the institutional attention that data science has attracted and the power of some accounts in broadcasting data science ideas even in the confines of the small dataset.

**Figure 3:**
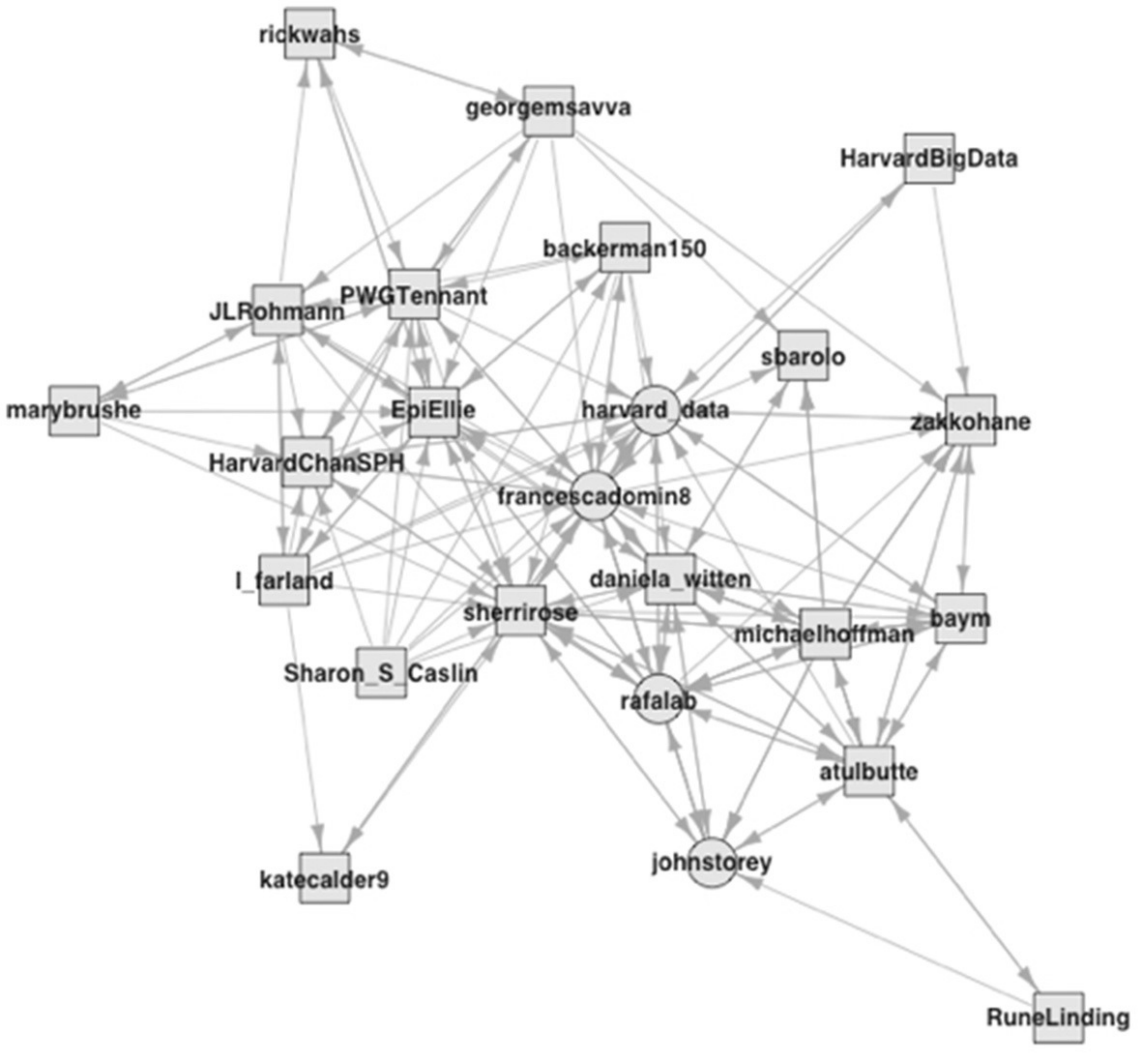
Followership relations within the biostatistics group (2).

The second group consists of 24 accounts (Figure 3), which capture a different side of the community, and one with more interconnections than the previous group at a density of 0.31.^12^ There are few, if any, broadly familiar accounts, which mostly belong to epidemiologists and biostatisticians. We see accounts with Harvard affiliations, but this time, they belong to a data initiative and the public health school. Most of these accounts are, again, second-degree neighbors who have entered the observations via direct connections, which are central in this group. The public prominence of media accounts ensured the diffusion of their tweets in the first group. In contrast, this group's academic culture of communicating knowledge and ideas contributed to their diffusion beyond a tight boundary. As these accounts entered the analysis via data science-related tweets, they reflect the idea that expert work unfolds in problem areas rather than formal groups (Abbott, 1988).

Table 2 presents the structurally most central actors of cluster three, which is too large to show visually (it consists of 115 accounts). This group is quite tightly interconnected, considering its size, with a density of 0.15. The most central first-degree neighbor account belongs to hadleywickham, a former professor of statistics, developer of popular R packages, and now a research scientist at RStudio, a software company with free software options. There is also seanjtaylor, who introduced himself on Twitter as a research scientist at Lyft at the time of this analysis but has used the data scientist label for his roles in the past and has continued commenting on data science issues. Another central account is robinson_es, who introduced herself as a data scientist at Warby Parker and advertised a book on building a data science career in her Twitter bio. The most central second-degree accounts are similar, with JennyBryan as a former professor who is now with RStudio, like Wickham, or skyetetra, who introduced herself as a data scientist and author of a book on data science careers, like robinson_es. While not all are equally technical, they all work with data, both first- and second-degree accounts. We can think of this group as data hackers and potentially the group that fits the opening definition of data science most closely. The dominance of second-degree neighbors in this institutionally undefined group of technical profiles indicates the relational backbone of data science's construction.

**Table 2:** Overview over 15 most central accounts in the hacker group (3).

	**First-degree accounts (n**=**36)**			**Second-degree accounts (n**=**79)**		
**Rank**	**Screen name**	**Followers**		**Screen name**	**Followers**	
		**Sample**	**Twitter**		**Sample**	**Twitter**
1	hadleywickham	80	102,274	JennyBryan	60	31,404
2	drob	63	42,386	CMastication	54	11,764
3	seanjtaylor	63	28,795	minebocek	53	10,980
4	hspter	58	26,384	kara_woo	51	9,152
5	vboykis	55	22,158	beeonaposy	48	11,089
6	robinson_es	54	20,391	thomasp85	45	18,278
7	KLdivergence	40	8,919	noamross	42	7,195
8	thosjleeper	40	8,240	brookLYNevery1	38	4,910
9	kierisi	39	10,652	skyetetra	37	6,669
10	Rbloggers	37	81,035	WeAreRLadies	32	17,833
11	_inundata	35	10,453	ChelseaParlett	32	10,985
12	DataSciFact	34	115,504	ludmila_janda	28	1,644
13	jim_savage_	31	6,584	bencasselman	17	59,604
14	sarah_guido	30	7,429	dan_p_simpson	16	3,469
15	thomas_mock	29	6,174	databozo	15	1,483
Summary	Mean	28	25,884		12	30,326
	Median	25	7,134		7	1,738

Summary statistics rounded to integers for clearer display.

**Figure 4:**
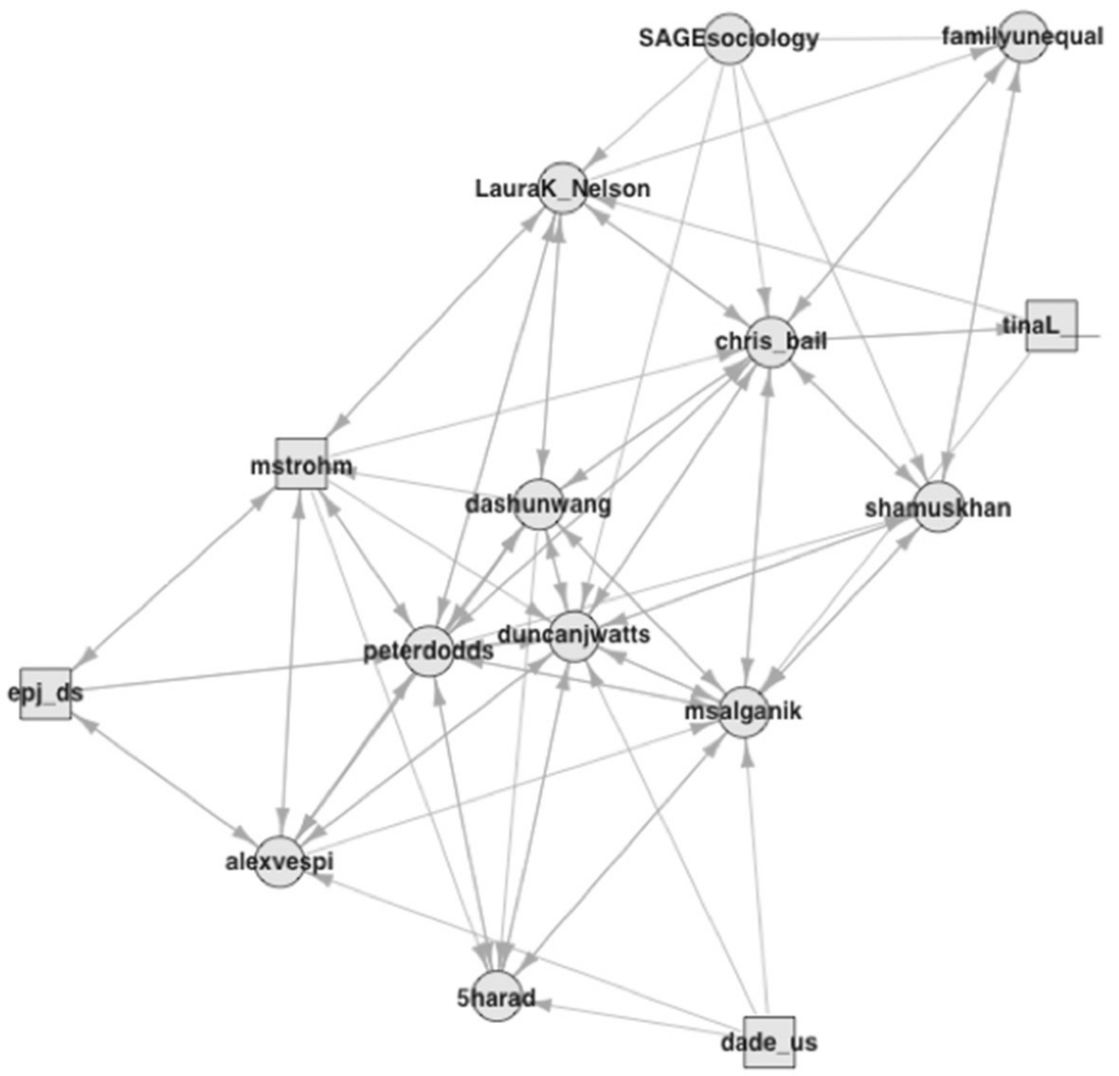
Followership relations with the social scientists group (5).

Consider, in contrast, the fourth group, which consists of only 15 accounts and contains some of the social scientists that have shaped data science (see Figure 4). The interconnections are strong, like in the other cluster of predominantly academic accounts, and have a density value of 0.39. The most central account among them belongs to Duncan Watts (duncanjwatts),^13^ now a professor at The University of Pennsylvania, following several years as a research scientist at Microsoft and as a sociology professor at Columbia University. During my field observations, I heard a story that quantitative analysts at Facebook, where the mythology locates data science's origin in the mid-2000s (Hammerbacher, 2009; Davenport and Patil, 2012), consulted Watts for advice on the label. Matt Salganik (msalgnaik), another central node, is a quantitative sociologist at Princeton University who wrote a book about quantitative research in the digital age that addressed both social scientists and data scientists (Salganik, 2018). Laura Nelson (LauraK_Nelson) is a sociologist at the University of British Columbia and promotes principles from qualitative methods for computational research (e.g., Nelson, 2020). Not necessarily well-known outside academic circles, all these scholars have apparent connections to data science. Shamus Khan (shamuskhan), on the other hand, does mostly qualitative research, but he has published quantitative studies as well (e.g., Accominotti et al., 2018). He appears in this dataset because he still tweeted about a data science opportunity at Columbia University, where he taught at the time. Following a media group, epidemiologists, and the hacker group, this is a social science group. The large share of first-degree neighbors in this group of social scientists amid its small size captures my own position in this analysis and suggests that social scientists are keeping quieter than they could about data science [see Ribes (2019) and Brandt (2022) on this issue].

The last group, cluster five, is also the largest (177 accounts) and has some of the nominally most explicit connections to data science. Table 3 once again focuses on the most central accounts out of another quite interconnected cluster, considering its size, with a density value of 0.15. The names may not be immediately familiar, but many of them participate actively in the advancement of digital tools. In contrast to the hacker group, this group often comments on broader issues and developments. hmason is the most central node among the first-degree accounts, consistent with her status as a data scientist, founder of a data startup, and co-author of an early data science definition,^14^ as well as a book on data science ethics (Loukides et al., 2018). AndrewYNg is a Stanford professor, co-founder of Coursera, and head of artificial intelligence at Alibaba. Then, there are also wesmckinn and amuellerml, who do quite technical work. There is KirkDBorne, formally the chief data scientist at Booz Allen Hamilton at the time and a data science popularizer, but also mathbabedotorg, who was a math professor before she became a data scientist and eventually an activist and author who points at issues with algorithms (O'Neil, 2016). The second-degree accounts mirror the direct neighbors, as for the hacker group, just trailing them slightly in centrality. Many have similar technical skills as those in group three, and several have PhD-level training, but they also bring weightier institutional affiliations, which makes them possible data science visionaries. The balance between two groups in this more talk- and thought-focused cluster shows the beginnings of data science as a distinct object.

**Table 3:** Overview over 15 most central accounts in the visionary group (5).

	**First-degree accounts (*****n*** = **80)**			**Second-degree accounts (*****n*** = **97)**		
**Rank**	**Screen name**	**Followers**		**Screen name**	**Followers**	
		**Sample**	**Twitter**		**Sample**	**Twitter**
1	hmason	118	122,176	hardmaru	60	81,974
2	AndrewYNg	110	470,649	PyData	51	49,007
3	dpatil	110	70,709	DataJunkie	47	20,030
4	BecomingDataSci	95	54,636	GaryMarcus	45	41,192
5	ylecun	94	184,725	_brohrer_	42	11,079
6	kdnuggets	92	167,719	DynamicWebPaige	40	32,677
7	wesmckinn	89	47,116	anildash	39	599,617
8	amuellerml	88	40,043	acroll	38	25,228
9	jeremyphoward	86	92,563	DiegoKuonen	35	22,340
10	KirkDBorne	85	256,193	mikeloukides	35	6,842
11	peteskomoroch	85	47,010	SciPyTip	35	94,272
12	chrisalbon	85	45,544	samcharrington	31	16,544
13	drewconway	83	24,817	skamille	30	30,703
14	randal_olson	76	124,188	jeggers	26	8,796
15	kaggle	75	173,799	tianhuil	26	4,756
Summary	Mean	42	38,959		12	15,892
	Median	35	12,771		7	1,547

Summary statistics rounded to integers for clearer display.

The network's fragmentation into five groups in the small dataset captures the distributed organization of the data science conversation. It reveals the technical and popular perspectives in data science as well as potential sources for non-technical ideas and my social scientific perspective. The first analysis of the large dataset suggested a simple picture that reproduced the familiar divisions. It captured the larger divide between technical expertise and general issues in which data science flourished but not its micro-level foundation. The second analysis of the small dataset revealed fragmentation of the accounts followership network into groups that are internally plausible and reveal a more complex relational underpinning of data science's construction on social media, which involved some densely connected communities that still tied into neighboring groups. The two analytic lenses complement each other to indicate a fractal structure (Abbott, 2001). This additional complexity shows the counterintuitive implications of accounting for “the larger social world” and its promise for studying an emergent group. The different group compositions have started suggesting different motives for data science's definition. The next two steps study them directly.

### 4.2 Purposes

This step turns to purposes to move further toward a Burke-informed cultural understanding of data science's construction on social media from Mohr's computational hermeneutics perspective. Twitter users can indicate a tweet's purpose through hashtags, and popular hashtags in a group indicate the group's purposes. This step analyzes the prominence of different hashtags using weighted log odds ratios. Odds ratios in text analyses measure the odds for a word occurring in one corpus compared to another (Silge and Robinson, 2017), such as in speeches by Republicans and Democrats or in tweets in the small and large datasets. The frequency of words in two corpora may vary vastly, and they do so by design in the large dataset of missed tweets and the small dataset of qualitative observations. Log odds ratios correct for these asymmetries, but words that do not occur at all in one corpus remain problematic. The following analysis uses weighted log odds ratios, which account for words that may have occurred by chance (Monroe et al., 2008; Schnoebelen et al., 2020).^15^

**Figure 5:**
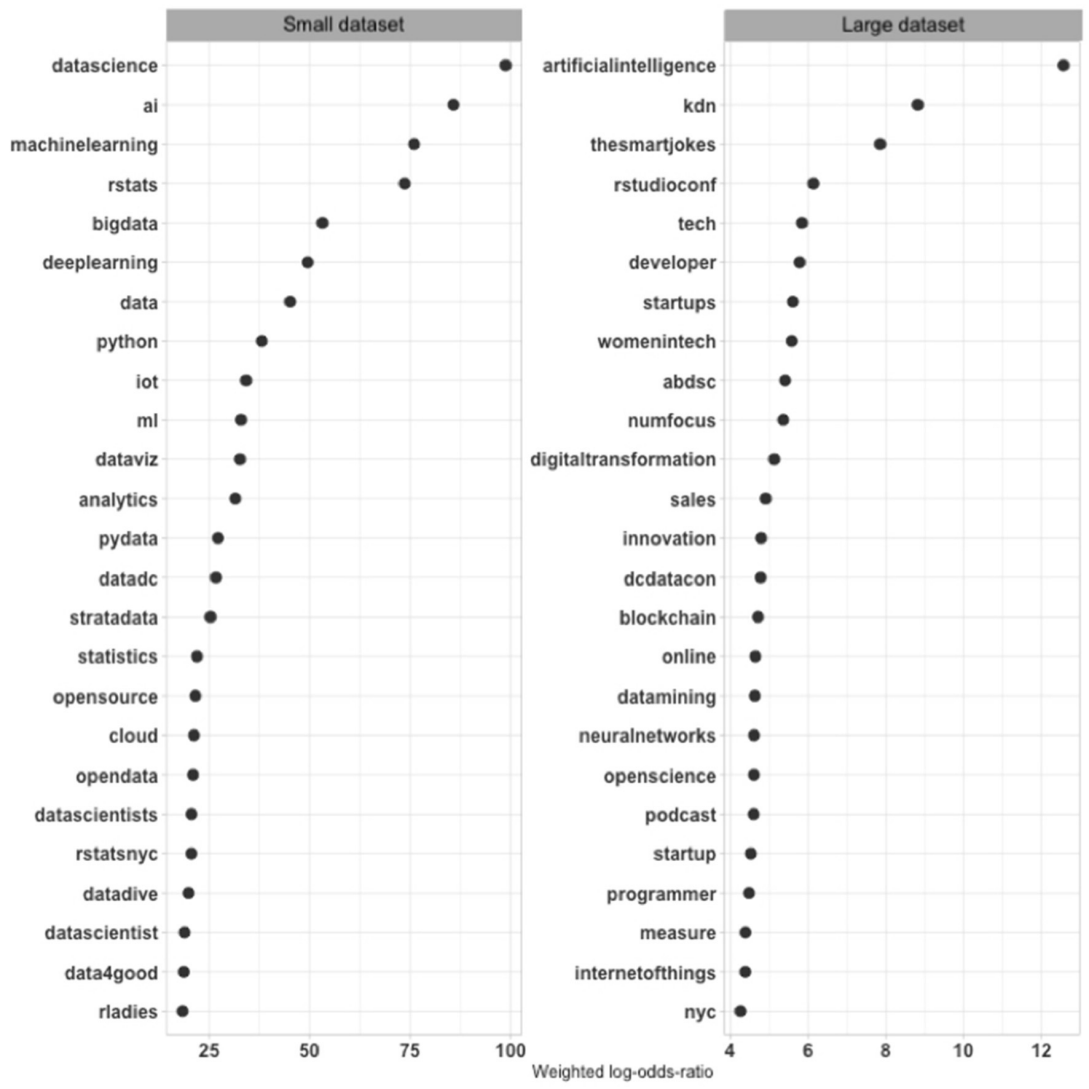
Weighted log odds ratios of hashtags in small dataset and large datasets.

This step starts once again with the most comprehensive dataset. The tweets in the large dataset include 335,337 hashtags (46,971 unique hashtags). Figure 5 shows the 25 hashtags with the highest weighted log odds ratios from the large corpus compared to hashtags from the small tweet dataset. The large one includes tweets that promote technical and commercial concerns through hashtags such as *artificialintelligence, neuralnetworks*, which operationalize artificial intelligence, and *internetofthings*, on one side, and *startups* and *innovation*, on the other. *nyc* was promoted as well, reflecting the location of the qualitative observations but also its significance in broader discourse, as were women in tech. The *blockchain* hashtag captures broader technology purposes among these tweets. These are big issues and a range of different ones. Consistent with some of the existing writing (O'Neil, 2016; Eubanks, 2018; Zuboff, 2019), data science and related concerns thus emerge as part of a comprehensive effort, or a larger cultural discourse, to promote technology and business, the large corpus shows.

Similar to the initial community structure, these are reflections of familiar purposes of technology and data science advocates. Their occurrence in the tweets dataset underlines Twitter's utility for studying data science's construction, but the bird's-eye view offers few new insights. Next, I turn to the small dataset.

The small dataset includes 475 hashtags (213 unique hashtags). The list of hashtags with the largest weighted log odds ratios on the side of the small dataset includes several that directly or indirectly promoted data science, such as *datascience, data, bigdata, AI, ML*, and technology themes, such as *python, pydata, rstatsnyc*, and *rladies*. The hashtags *rladies* and *data4good* promoted political and moral purposes, similar to some prominent purposes in the large dataset but with different political connotations and more concrete initiatives. Some of the hashtags stand for groups or conferences, such as *strataconf* and *datadive*. *datadive* described events where a group meets to work closely on a dataset, while *strataconf* referred to a major data conference with expensive tickets. *rstatsnyc* captured the promotion of a local community and reflected the new hope that New York gained as a tech location vis-à-vis Silicon Valley in the latest technological transformation. The hashtags that capture local or topically specific purposes show the payoff of taking different perspectives and moving to a smaller dataset. Twitter facilitates global discussions, but it also accommodates local ones, and they are potentially crucial for mobilizing support and involvement.

The distinctive hashtags reflect purposes that start revealing data science's roots in a collective project around technical skills and ideas for a professional community. The technical hashtags are not distinctive for data science, however, as critics have often noted. The hashtags that stand for community activities, which are not part of the popular data science discussion, suggest a process wherein diverse technologies gain a joint meaning as data science.

**Figure 6:**
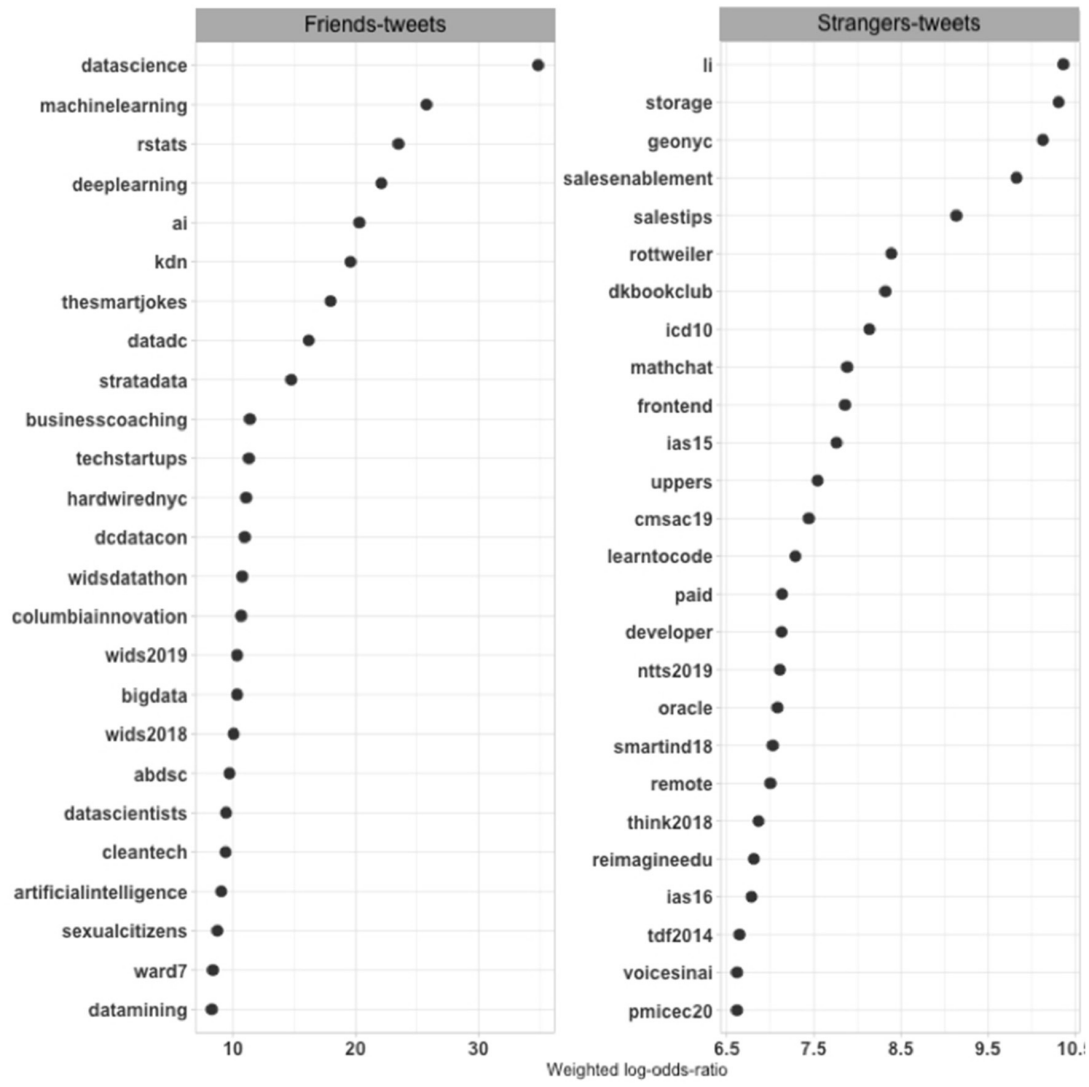
Weighted log odds ratios of hashtags in tweets of first- and second-degree accounts in the large dataset.

The contrast between the large and small datasets serves as a necessary first step to establish the utility of this approach but may overlook variation from more gradual shifts of perspective. One complementary step compares purposes associated with second-degree accounts to those of the first-degree accounts within the large dataset of missed tweets (see Figure 6). Tweets by second-degree accounts included 186,607 hashtags (36,131 unique hashtags), and tweets by direct neighbor accounts included 148,718 hashtags (17,291 unique hashtags). Some outlier hashtags appear on these lists.^16^ Purposes are once again more diffuse across second-degree tweets in the large dataset. They include *oracle*, which is a database firm and synonymous with that firm's technology, and storage, referring to data storage that data scientists have relied on from early on (Hammerbacher, 2009), and *voicesinai* or *learntocode*—other technical concerns. Then, there is more on sales and several hashtags that promote different technical conferences in the late 2010s. New York City features again as well.

The first-degree accounts tweeted about a combination of the issues that appeared in the small dataset and the large dataset. Data science again tops the list, with machine learning and artificial intelligence nearby and R not far behind. w*ids2018* and 2019 appear on this list, promoting women in data science in general and a conference that Stanford University hosts for this purpose, an initiative that has spread to a large number of institutions. This list still includes more of the commercial concerns that the small dataset missed, such as *techstartups* and *businesscoaching*.

The differences between first-degree and second-degree purposes remain smaller than between the small and large datasets to capture a more continuous view of the different levels and contexts of data science's construction on social media. The small dataset systematically reveals locally and topically specific purposes that connect the purposes data science supporters share more generally to the situations of specific supporters or beneficiaries. Overall, the small tweet dataset captured most clearly the promotion of data science issues, even in technical terms, and collective activities that would be part of data science's “cultural machinery” (Abbott, 1988, p. 60). Together, the different perspectives captured how new socio-technical arrangements come together in expert work (Eyal, 2013). The purposes across the large tweet dataset spoke to broader tech and business concerns, reflecting the larger cultural shifts of the digital era. These purposes, missing from the small dataset, were more prominent among second-degree accounts than among direct neighbors. Instead of constraining the analysis to a representative picture, the comparisons capture the “larger diversity in the world” (Krause, 2021) at varying depths of data science definitions.

### 4.3 Scenes

The final analytical step turns to “scenes” to see the contexts wherein the actor groups articulate purposes (Burke, 1945, p. 3) as part of their construction of data science on social media. Mohr et al. (2013) used latent Dirichlet allocation (LDA) topic models for recovering scenes from texts, which identify words that co-occur in documents within a larger corpus of documents. Each word may be part of one or more topics, and each document may consist of one or more topics (Blei et al., 2003). Several specialized topic modeling approaches are available for specific research problems. This analysis follows Mohr's approach and uses LDA topic models “to identify the lens through which one can see the data most clearly” more than “to estimate population parameters correctly” (DiMaggio et al., 2013, p. 582). In this sense, the following models provide an initial image of data science's cultural construction while tracing its contours from varying perspectives.^17^ They treat tweets as documents after removing hashtags, addressed accounts, URLs, stop words and numbers, and use word stemming.^18^ Consistent with the earlier steps, I generated separate topic models for the large dataset of missed tweets and the small tweet dataset and, within the large dataset, for the tweets of first- and for those of second-degree accounts. This division into distinct corpora captures the scenes as fresh looks from each of the perspectives, revealing their misses, and gains. Computational limitations demanded taking samples of 35,000 tweets from the large dataset of missed tweets for each of the three analytical steps.^19^

The first step starts again with the large tweet dataset of full timelines missing from the small dataset. The analysis revealed 45 topics, of which many have no connection to data science, reflecting that it was not a strategic endeavor and instead part of the much broader conversation on Twitter, but data science-related topics still emerged even in this bird's-eye view. Overall, ten topics were about data science issues, another ten about tech or science issues, and then nine, six, and ten about current issues, mostly politics, miscellaneous topics, and different types of chatter (see also Table 4).

**Table 4:** Summaries for topic models of large tweet dataset.

**Perspective**	**# of topics for three topic models**					
	**Data topics**	**Tech topics**	**Current issues**	**Misc. topics**	**Chatter**	**Total**
All	10	10	9	6	10	45
First degree	7	11	8	4	15	45
Second degree	13	12	6	3	6	40

The tech and science topics comment on the digital transformation, for example, startup opportunities and the big technology companies, as well as articles and journals that are relevant to these accounts. The topics that capture discussions of generally important issues include topics around Trump and politics, education, the economy, and healthcare, as well as urban and civil rights issues. Then, there is a group of leisure topics, including sports, movies, and music, cultural concerns in the lay sense. Finally, several topics have no specific substantive meaning and instead reflect observations, opinions, pleasantries, and general Twitter chatter.

As Supplementary Table S1 shows, the data topics captured quite a few dimensions of data science, a striking result considering the simple modeling procedure, diverse accounts, and openness of Twitter as a discursive space. More specifically, data topics cover practical issues, such as careers and hiring, but also training and studying. The more technical among them revolve around different data analytic approaches or procedures, ranging from statistics and causal inference to machine learning and artificial intelligence, as well as coding-related issues or data visualizations. Perhaps most interestingly, this analysis revealed a topic that picked up on issues of bias and ethics. These topics cover the dimensions of data science that are familiar from more formal, deliberate, and curated discussions directly from concrete conversations. They still present a mirror image of the familiar themes of data science discussions. This broad view responds more to data science rise than its meaning construction, which the small dataset was designed to capture.

The tweets in the small dataset cover 13 topics or, in Burke's terms, scenes. Table 5 lists these topics as 20 words most closely associated with each of them. The table also lists names that I assigned to topics as summaries. Topics two (2) and 13 may be labeled statistics and machine learning. Topic 2 includes words such as *model, logistic, regression*, and *algorithm*, and topic 13 includes *machine, learning, code*, and *python*, a popular programming language. Topic 11 is about software issues and their importance for data science, several words suggest. Topic seven (7) seems to discuss data science relative to other roles, and topics nine (9) and ten (10) include career advice and open positions. Topic four (4) describes data science training, which seems essential if topic three (3) is right about the challenges it indicates. The tweets associate successful data science with team efforts, as topic six (6) suggests. Topics five (5) and twelve (12) capture discussions and exchanges at conferences and in digital formats as other scenes.

**Table 5:** Thirteen level topic model of small tweet dataset.

**Topic**	**Label**	**20 words associated with topic**
1	REFLEXIVITY	say, compani, case, win, type, realli, room, challeng, word, happi, notebook, hour, effect, get, like, creat, anoth, divers, jupyt, true
2	STATS	model, use, algorithm, learn, statist, call, think, just, rare, regress, someth, bad, chart, non, take, logist, new, wow, can, motiv
3	CHALLENGE	think, work, problem, right, don, good, know, deep, anyon, solv, experi, sure, project, often, mani, use, need, tool, prepar, without
4	TRAINING	data, scienc, program, new, appli, social, cours, student, work, univers, interest, statist, applic, hire, posit, research, school, depart, human, phd
5	TALK	data, scienc, talk, great, good, build, nyc, speak, thank, confer, first, ever, kaggl, communiti, best, tool, lot, industri, convers, podcast
6	TEAM	team, new, support, join, help, work, great, look, amaz, scientist, way, come, thank, year, awesom, communiti, person, product, grow, use
7	ROLES	data, scienc, scientist, time, peopl, compani, job, work, statistician, career, engin, interview, question, one, role, ask, post, mani, see, lot
8	PURPOSE	better, read, just, differ, seem, like, interest, ethic, much, paper, mean, someth, process, thought, part, discuss, nice, class, design, place
9	CAREER	data, scienc, will, first, scientist, year, day, start, career, book, time, open, make, now, today, announc, new, project, excit, big
10	POSITION	data, scientist, scienc, need, team, facebook, can, now, read, make, core, don, take, chief, compani, hire, miss, set, ethic, everyon
11	SOFTWARE	can, engin, import, one, also, make, softwar, thing, just, even, come, see, code, still, field, relat, say, well, feel, kind
12	SOC.MED.	get, post, know, follow, thank, peopl, can, look, will, want, blog, make, tell, think, give, done, write, time, respons, share
13	ML/RES.	data, learn, scienc, scientist, want, just, use, machin, one, can, code, don, like, get, help, articl, real, thing, python, now

ML, machine learning; RES, research.

These topics reveal a more refined set of scenes that still show analytically important depth and diversity. The scenes are familiar from the popular data science discourse, and they reflect themes from sociological ideas about expert work. Several books describe the technical challenges associated with data science work (e.g., Schutt and O'Neil, 2013; Wickham and Grolemund, 2016), universities have started to offer data science training (Börner et al., 2018; Saner, 2019), data scientists have discussed their roles and careers (Shan et al., 2015), and how to build teams (Patil, 2011). The concern with neighboring roles echoes Abbott's classic idea about conflicts between expert professions (Abbott, 1988). The overlap between existing contributions, topics from the large dataset, and this collection of tweets gives confidence in the utility of a small dataset for analyzing data science's cultural definition on social media. In contrast to the existing contributions, these topics portray scenes of ongoing development requiring concrete engagement rather than definite frames of reference and larger processes.

However, the first topic (1) seems neither intuitive nor familiar. Some words are clear enough: Data scientists often work in companies, for instance, while *challenge, win*, and *happy* may also go together, as data analysis competitions are a popular sport and recruitment tool in data science. *say, word, hour*, and *room*, in contrast, make less intuitive sense. A topic modeling approach provides the opportunity to deal with such surprising results by returning the documents that included these words (e.g., Karell and Freedman, 2019). Some tweets were about an analysis of gender diversity that won a data challenge; others discussed the diversity of data scientists in the room should reflect the outside world. Authors of further tweets wondered what they should say to their audience in a room during the half-hour that they had to speak to them. Topic eight (8) echoes the reflective ideas behind these issues. It consists of words that suggest these users reflect on broader problems, including *ethics, discussion, thought, read*, and *better*, but the first topic insists on recognizing the collective challenges around advancing these issues as part of data science, adding substance to the conference-related purposes in the previous analysis.

Like the other topics, the reflective perspective has appeared in the broader discourse (O'Neil, 2016), and some of these tweets may concern proposals for a code of ethics for data science (e.g., Loukides et al., 2018). These observations capture the collective discussion of these topics and the original implications for active data scientists. Again, however, the general ethic topic manifests itself in discussions of practical questions about implementing it in the community. The initial ambiguity about the words in topic one captures the close connection between these generally familiar ideas and the real experience of constructing a novel professional role.

The final comparison reiterates the analytical strategy of comparing a wider perspective to a narrower one without the radical difference between the full large dataset and the small dataset. It compares topic models of corpora from subsets within the large dataset of missed tweets of tweets of first- and second-degree accounts, which remain closer to the project's theoretical focus.^20^ Similar to the full large dataset, these models revealed 45 and 40 topics, which I once again report in thematic groups. Table 4 presents the summaries (together with the full dataset as an additional reference); Supplementary Tables S2, S3 show all topics in terms of their top 15 words.

Like the initial model for the large tweets dataset, these models reveal familiar scenes and additional ones that the small dataset missed and a more refined set of these topics from tweets by first-degree accounts than in the second-degree tweets. The different groups map onto those from the initial description, with some details that I discuss below. More interestingly, the shifting perspective shows, again, benefits for locating data science's construction in its larger social context. The slightly broader perspective focusing on second-degree tweets has much fewer topics focused on data issues and, to a lesser degree, on tech and science, and more on current issues and especially general social media chatter. While they do not have an evident connection to data science's construction, they serve as an important indicator of where that construction happened, namely, among general concerns and not only the specialized scientific concerns that were more salient in the network analysis.

The dataset of missed tweets by first-degree accounts already reveals a more refined set of data-related topics as well as reflexive discussions. It includes an ethics topic, reflecting this issue's prominence in data science discussions and the well-documented strategy for gaining legitimacy (Abbott, 1983). Here, ethics appear in the context of algorithmic bias, which is part of the larger conversation. In the small dataset, in contrast, the diversity concerns appeared as well around the problem of discussing it in the data science community and its audience and self-reflection on recognizing the purpose of the data science role. Both ethics scenes, in the large and small datasets, are about non-technical questions about what is right, but they differ on how this concern presents itself to those who confront the scene.

The asymmetric comparison shows the limits of the each dataset for capturing meaning construction. Shifting perspectives to narrower dataset designs reveals locally meaningful scenes of concrete engagement with the collective construction of data science as a social object. This pragmatic reflexivity from the small dataset remained largely absent from the larger datasets. The analytic strategy then indicates the utility of considering different levels of data science's cultural construction instead of settling on one definite level for studying an emergent process, especially one that seeks the largest possible view. It also points to technical directions for implementing a more refined text analysis that considers immediate word contexts on the large dataset that tests ideas following from the small dataset.

## 5 Discussion

This analysis departed from a limited perspective to gain analytical traction on data science discussions on social media from a cultural perspective, an emergent process that poses unique research design challenges that today's digital affordances can help address. Initial examples of tweets illustrated reflections of an emerging profession around technical knowledge, training, and jobs, as well as the wider digital change. The results of network and text analyses found patterns consistent with existing research on data science, as well as ideas in the literature on expert work and quantification. They extend recent arguments that data science's emergence follows from an ambiguous image in its outside construction in firms and sciences (Dorschel and Brandt, 2021) and the struggle of individual data scientists with that ambiguity (Zuboff, 2019; Avnoon, 2021). This analysis captured how the data science community sorted out that ambiguity on social media. The qualitative research on which this study built identified meaning-making around concrete analytical and relational issues. This computational ethnography showed that data science pioneers reflected on these challenges between each other and how they arrived at the specific issues in more general discussions.

The analysis addressed the research design challenge of studying emergent processes by adopting an “active approach to data” (Leifer, 1992). It integrated ideas from qualitative and quantitative research about missing observations to guide an analysis of two complementary datasets in an asymmetric comparison (Krause, 2021). This comparison captured the interplay of how actors integrate broader cultural shifts and their more technical ideas into a novel professional identity. Instead of resorting to a single scope or boundary, this article makes an argument for using computational tools to gain analytic leverage from the variation across different boundary specifications. For quantitative analysts, this approach means that rather than departing from the idea of a general analysis, which has merit in many situations but works less well for capturing localized meaning-making processes (e.g., Nelson, 2021), they can approach a research problem in relation to their point of departure and comparing different angles on a specific case or process. This approach offers one solution to the increasingly important question of the relevant scope of quantitative analyses (Lazer et al., 2021).

These conclusions are subject to limitations. Subsequent research has to establish connections between the scenes and purposes and the actors for better understanding data science's development. This article's focus on the emergent moment and the methodological challenges that come with it benefited from relying on basic network and text analytic procedures. They can serve as points of departure for analyses that discover more nuanced social and meaning structures. More advanced social network analysis techniques can untangle the precise attachment processes between accounts, such as between the groups this initial analysis reveals. Similarly, more advanced text analytic techniques can identify more nuanced topics and meaning changes of words, such as around the technical and non-technical issues this analysis revealed. More broadly, additional studies of data science have to step outside the Twitter setting to consider agency and acts, but these findings also invite research on further professional or otherwise collective activities on Twitter and how they use social media to discuss with each other in public.

Keeping those limitations in mind, these insights into the collective definition of a professional role complement existing views on professions of expert workers defending their boundaries against competitors (Abbott, 1988), establishing themselves in modern corporations (Muzio et al., 2011), or navigating more extensive socio-technical arrangements (Eyal, 2013). The analysis revealed actors outside of broad commercial and narrow technical concerns, a potential source of new views, and a distinct motivation behind starting data science: building a platform to adopt new practical and ethical standards. While familiar from other scientific and intellectual movements (see Frickel and Gross, 2005), this motive appears here for the first time for data science. Compared to other professions that acknowledge non-technical aspects of their work (e.g., MacKenzie and Millo, 2003), data scientists discuss these concerns as a community, integrating them into their stock of knowledge.

Practicing data scientists can use this glimpse into their early days as a reference point for assessing their current situation and future direction as a profession. The digital era renders the institutional scaffolding of classic professions less necessary for collective organizing (Avnoon, 2023). This advantage does not relieve professionals from mutual engagement over the content and contours of their work if they seek autonomy from their employers. More immediately, data scientists can also find utility in the culturally informed computational analysis and design around qualitative approaches.

## Data availability statement

The raw data supporting the conclusions of this article will be made available by the authors, without undue reservation.

## Ethics statement

Ethical approval was not required for the study involving human data in accordance with the local legislation and institutional requirements. The social media data was accessed and analyzed using the Twitter API in accordance with the platform's terms of use and all relevant institutional/national regulations. Written informed consent was not obtained from the individual(s) for the publication of any potentially identifiable images or data included in this article because only information participants chose to share publicly on Twitter was used for the analysis.

## Author contributions

PB: Conceptualization, Data curation, Formal analysis, Investigation, Methodology, Visualization, Writing – original draft, Writing – review & editing.

## Footnotes

^1^ https://twitter.com/josh_wills/status/198093512149958656

^2^Newspapers regularly cite data scientists as sources in or protagonists of their stories, and data scientists have featured in popular culture such as in Netflix's *House of cards* (seasons four and five).

^3^Twitter and the interface have gone through substantial change, even before the Elon Musk takeover and its rebranding into X. This analysis focuses on a relatively short window, however, and within that window on a specific corner of the Twitter discussion. The stability assumption is robust within that scope.

^4^See Alexander et al. (2012) for this reflexive view on computational hermeneutics.

^5^The large dataset missed tweets because Twitter only grants access to a given account's 3,200 most recent tweets. Potentially problematic for some purposes, the over seven hundred thousand available observations offer important context to the small dataset.

^6^This tweet was from January 2018, before revelations of MindGeek benefited from videos posted without consent. While such a tweet would indicate ignorance today, at the time it more likely tried to present a progressive twist on possible areas of professional work.

^7^I identify individuals by name if they maintained a public profile in the community.

^8^While this number looks large, it only represents 8% of all possible ties. In addition, 13 accounts did not follow any accounts and remained outside of the network.

^9^There was a sixth group with only three accounts as well as 15 isolates and two isolated pairs that I leave out of this description.

^10^Density is a social network analysis measure that indicates the share of all ties in a network out of all possible ties with 1 as the highest score.

^11^I refer to the Twitter account names since they serve as the main method for using Twitter and what users have chosen to share as their public profiles.

^12^The density measure is sensitive to networks of different sizes in terms of numbers of nodes. In this analysis, the similar density scores between this group and the media group despite their vastly different sizes highlight the great importance of direct following relationships in this group.

^13^I report the names together with Twitter usernames for this group because the accounts belong to social scientists and may already be familiar to readers.

^14^ https://web.archive.org/web/20160220042455/dataists.com/2010/09/a-taxonomy-of-data-science/

^15^A related measure with similar qualities is the tf-idf measure. The weighted log-odds-ratios capture better words that are common in different corpora but still more salient in one than another, which is important for this analysis that compares different perspectives.

^16^*li* and *rottweiler* were outliers in tweets of second-degree accounts. One promotes the account itself and the other the account owner's dog amid other tweets about data science issues, indicating personal promotion efforts. On the friends side, the *sexual citizens* hashtag does not fit with data science. It refers to a book that had been recently published by Shamus Khan, one of the academic friends accounts, together with Hirsch and Khan (2020). This hashtag also promotes a personal project, a book, that has a collective orientation at the same time. This difference indicates that the project's interest in data science's collective construction may have led to overlooking actors who pursue more self-serving purposes, supporting the benefits of the asymmetric comparison. As both agendas appear systematically, data science may not have a uniform definition at this early stage.

^17^The more specialized implementations can account for meta information on the documents for estimating topic models. At this initial research step focusing on the effect of different perspectives on the emergent image of data science, no specific meta information informed the topic estimation. The discussion will outline how this study's results inform such more refined implementations in future research.

^18^I used the topicmodels package (Grün and Hornik, 2011) in R with the Gibbs sampler method and an alpha of .1. I obtained the number of topics after testing a series of possible numbers of topics using the ldatuning package (Nikita, 2020) and considering the four evaluation metrics the packages provides, particularly Griffiths and Steyvers's (2004). My qualitative reading of the results and familiarity with the case confirmed that this implementation provided satisfactory results for the purposes of observing data science's construction across the different perspectives.

^19^This limitation only has small effects on the results. While topic models of more tweets obviously capture more topics (in contrast to other many other corpora, Twitter specializes in no particular set of issues), analyses of different sample sizes and randomly composed corpora have revealed the same set of main topics.

^20^Specialized techniques exist [e.g., correlated topic models (Blei and Lafferty, 2009)] for modeling these two corpora jointly while considering the two types of accounts. Rather than finding the one precise topic model, however, this analysis aims to compare the “lenses” different points of departure offer.
