# Supplementary material for: Data science's cultural construction: qualitative ideas for quantitative work
Source: Front Big Data. 2024 Aug 14;7:1287442. doi: 10.3389/fdata.2024.1287442 (PMC11349665; doi:10.3389/fdata.2024.1287442)
Supplement: Supplementary file 1 [file Table_1.DOCX]

| Supplementary materials  Table S1 | |
| --- | --- |
| 45 level topic model of large tweets dataset | |
| Topics | Top 15 words |
|  | Data topics |
| 1 | free, cours, data, now, sign, onlin, today, learn, scienc, live, week, get, avail, regist, seri |
| 7 | model, use, network, languag, neural, process, can, train, comput, natur, text, scale, system, larg, generat |
| 10 | can, system, make, design, bias, decis, need, ethic, algorithm, public, understand, help, tech, data, power |
| 13 | statist, use, social, result, model, network, differ, effect, predict, measur, studi, method, experi, infer, causal |
| 14 | new, intellig, data, industri, via, technolog, product, digit, cloud, artifici, busi, use, analyt, manag, report |
| 18 | job, data, research, work, hire, appli, scienc, look, engin, interest, team, new, program, softwar, applic |
| 23 | open, use, code, python, sourc, packag, tool, new, version, write, now, project, develop, creat, can |
| 24 | learn, machin, deep, use, python, new, via, build, imag, step, resourc, want, great, guid, start |
| 33 | data, scienc, scientist, use, big, analysi, visual, engin, project, social, collect, open, analyt, tool, good |
| 34 | use, function, just, type, number, differ, plot, one, chart, two, can, map, line, show, creat |
|  |  |
|  | Tech/Science topics |
| 2 | chang, tech, compani, big, world, will, busi, climat, way, custom, take, success, startup, citi, market |
| 15 | can, problem, get, make, better, way, work, find, peopl, help, much, solv, life, real, need |
| 19 | googl, app, use, search, new, appl, get, now, amazon, featur, facebook, just, mobil, run, websit |
| 20 | new, post, blog, check, latest, link, read, write, interest, report, thank, project, follow, great, york |
| 25 | thank, work, great, help, excit, team, amaz, communiti, proud, see, support, project, friend, come, make |
| 28 | talk, video, listen, slide, check, podcast, interview, episod, present, watch, discuss, great, youtub, now, record |
| 29 | paper, review, read, research, publish, articl, journal, work, interest, thought, public, author, issu, report, recent |
| 37 | will, pay, peopl, money, cost, get, million, compani, make, employe, need, fund, hous, high, price |
| 44 | twitter, question, tweet, pleas, can, follow, email, answer, peopl, get, send, give, help, ask, thank |
| 42 | join, talk, confer, event, will, come, open, now, host, speaker, next, meetup, excit, announc, tomorrow |
|  |  |
|  | Current issues topics |
| 4 | trump, vote, presid, will, elect, parti, say, hous, polit, state, senat, democrat, candid, aign, media |
| 16 | health, care, medic, public, new, patient, cancer, studi, doctor, drug, mental, help, research, healthcar, diseas |
| 22 | news, case, world, may, use, death, live, report, fake, coronavirus, break, confirm, offici, spread, china |
| 26 | state, number, work, increas, rate, low, per, level, peopl, energi, incom, higher, sinc, tax, high |
| 27 | citi, street, park, plan, home, san, nyc, map, love, bike, one, francisco, amaz, road, south |
| 35 | women, black, peopl, men, white, gender, say, chang, studi, woman, face, mani, cultur, sexual, like |
| 38 | human, new, drive, car, test, take, robot, will, space, self, futur, bring, planet, via, air |
| 41 | law, polic, govern, rule, countri, american, fight, right, protest, peopl, state, war, nation, ban, gun |
| 45 | student, school, class, math, award, teach, univers, colleg, congratul, grad, research, educ, phd, program, high |
|  |  |
|  | Misc. topics |
| 3 | day, will, come, week, see, get, happi, today, hope, start, soon, next, readi, final, tomorrow |
| 11 | watch, show, love, art, movi, stori, star, video, math, rock, also, second, music, well, made |
| 36 | book, read, one, best, first, list, stori, ever, write, thing, just, written, year, favorit, will |
| 39 | game, play, team, win, competit, world, will, season, congrat, player, see, part, sport, chanc, footbal |
| 40 | like, look, forward, realli, feel, good, sound, cool, see, seem, littl, way, bit, just, much |
| 43 | food, eat, like, coffe, dog, good, look, two, drink, cat, order, shop, walk, bar, also |
|  |  |
|  | Chatter |
| 5 | time, get, long, everi, day, take, yes, work, need, tri, one, way, start, just, back |
| 6 | think, don, peopl, like, work, thing, can, mani, make, doesn, just, lot, realli, want, bad |
| 8 | think, peopl, thing, one, question, need, good, right, can, don, isn, much, know, like, even |
| 9 | year, last, time, one, ago, week, first, day, month, two, today, got, next, just, back |
| 12 | make, sure, thing, one, seen, just, know, haven, never, pretti, didn, yet, even, well, like |
| 17 | one, word, just, name, bad, can, use, good, also, thing, think, idea, realli, way, say |
| 21 | old, year, friend, live, kid, parent, get, now, stori, man, age, tell, life, never, famili |
| 30 | know, don, want, say, need, can, get, someon, anyon, els, let, like, just, see, everyon |
| 31 | thank, great, love, realli, good, see, much, share, hear, enjoy, interest, thought, talk, awesom, glad |
| 32 | now, right, can, just, keep, wait, back, close, take, happen, away, got, light, know, tell |

| Table S2 | |
| --- | --- |
| 40 level topic model of large tweets-dataset, tweets of first-degree accounts only | |
| Topics | Top 15 words |
|  | Data topics |
| 7 | learn, machin, deep, python, languag, model, part, use, practic, step, kdnugget, natur, guid, process, build |
| 11 | problem, can, make, design, need, work, use, solv, way, system, build, tool, product, focus, data |
| 12 | use, code, function, line, write, python, plot, like, packag, make, tri, type, panda, also, can |
| 13 | model, network, use, statist, test, distribut, method, random, predict, regress, bayesian, base, effect, infer, structur |
| 14 | now, free, data, cours, sign, today, week, miss, day, save, onlin, scienc, next, don, get |
| 15 | competit, win, kaggl, top, game, predict, world, challeng, just, launch, winner, award, best, congrat, team |
| 16 | data, scienc, scientist, team, job, engin, analyt, hire, big, busi, top, manag, skill, career, role |
| 17 | use, now, open, packag, code, sourc, python, releas, new, run, version, notebook, app, can, project |
| 25 | human, intellig, futur, artifici, via, can, comput, system, will, robot, say, connect, internet, valley, power |
| 28 | research, appli, scienc, data, open, social, comput, applic, work, hire, program, posit, look, interest, summer |
| 33 | data, use, analysi, big, visual, time, tool, seri, collect, explor, new, analyt, interact, predict, power |
| 35 | model, use, imag, network, train, neural, googl, base, paper, text, search, generat, languag, result, perform |
| 36 | bias, algorithm, ethic, law, tech, issu, human, gender, fair, decis, respons, system, need, public, power |
|  |  |
|  | Tech/Science topics |
| 4 | can, just, say, twitter, tri, email, get, got, tweet, don, know, use, someon, one, still |
| 5 | new, post, blog, follow, check, thank, york, read, first, share, comment, start, recent, link, peopl |
| 8 | work, thank, team, communiti, support, project, amaz, help, excit, great, make, love, proud, mani, today |
| 9 | paper, review, research, publish, read, journal, new, articl, issu, now, public, book, work, submit, author |
| 19 | think, differ, don, use, peopl, can, one, point, way, agre, also, mani, say, mean, thing |
| 20 | question, get, help, ask, can, want, answer, peopl, work, like, find, job, need, way, know |
| 23 | thank, interest, great, see, thought, work, also, read, nice, piec, articl, good, realli, thread, share |
| 24 | think, smile, social, media, facebook, news, twitter, polit, fake, group, inform, like, platform, user, account |
| 26 | compani, tech, busi, industri, new, startup, market, custom, big, product, servic, strategi, googl, cloud, platform |
| 27 | book, read, get, can, recommend, list, free, now, got, bit, start, page, best, need, write |
| 29 | talk, join, confer, event, excit, come, women, host, speaker, will, tomorrow, next, data, speak, see |
| 34 | talk, slide, video, watch, great, live, check, present, podcast, now, give, thank, record, episod, listen |
|  |  |
|  | Current issues topics |
| 3 | health, chang, world, new, care, climat, will, countri, global, person, diseas, life, real, via, risk |
| 30 | peopl, black, polic, protest, offic, just, kong, hong, like, fire, may, video, box, space, show |
| 32 | state, trump, vote, presid, elect, american, peopl, hous, will, say, countri, call, govern, white, democrat |
| 37 | student, teach, school, class, program, colleg, univers, educ, cours, high, studi, research, phd, success, math |
| 38 | citi, car, map, drive, area, bike, self, street, hous, come, road, park, public, stop, local |
| 39 | peopl, time, pay, will, cost, money, year, rate, women, famili, tax, mani, less, without, also |
|  |  |
|  | Misc. topics |
| 1 | look, thank, see, forward, great, happi, love, hope, day, good, today, time, meet, much, next |
| 2 | one, love, ever, made, name, stori, best, show, anoth, seen, first, play, word, behind, music |
| 18 | just, kid, food, eat, also, home, now, put, coffe, right, hot, hand, let, guy, water |
|  |  |
|  | Chatter |
| 6 | like, look, good, make, realli, feel, seem, thing, think, sound, just, lot, one, word, kind |
| 10 | day, time, hour, one, week, last, everi, can, get, first, still, minut, night, two, spend |
| 21 | know, don, peopl, think, can, want, need, like, work, thing, get, make, just, say, someth |
| 22 | will, time, world, get, can, one, like, work, better, thing, make, mani, take, place, live |
| 31 | just, well, good, yes, think, sure, right, work, can, much, one, probabl, even, done, still |
| 40 | year, time, ago, last, old, one, first, today, month, sinc, week, just, two, day, back |

| Table S3 | |
| --- | --- |
| 45 level topic model of large tweets dataset, tweets of second-degree accounts only | |
| Topics | Top 15 words |
|  | Data topics |
| 6 | use, code, python, packag, sourc, open, can, data, write, librari, tool, work, also, languag, just |
| 18 | learn, free, now, cours, onlin, get, check, code, machin, use, avail, train, just, tutori, start |
| 20 | data, scienc, scientist, use, big, analysi, work, visual, tool, good, analyt, set, collect, learn, check |
| 22 | learn, machin, use, model, deep, can, via, intellig, network, human, algorithm, train, artifici, imag, predict |
| 23 | data, new, report, cloud, manag, gigaom, market, analyst, technolog, product, analyt, busi, explor, discuss, next |
| 34 | model, data, use, can, test, effect, statist, result, method, analysi, measur, studi, think, valu, interest |
| 39 | can, use, line, number, list, one, point, chart, function, top, base, type, visual, way, nice |
|  |  |
|  | Tech/Science topics |
| 1 | paper, book, read, review, new, publish, best, journal, author, articl, list, interest, write, find, just |
| 3 | research, appli, open, project, new, program, applic, look, scienc, job, comput, now, hire, fund, work |
| 12 | join, confer, event, next, will, come, week, talk, tomorrow, day, now, excit, ticket, regist, session |
| 15 | team, help, build, work, can, product, compani, manag, experi, make, better, need, skill, custom, develop |
| 21 | tweet, twitter, email, pleas, can, follow, just, will, send, get, see, account, week, use, name |
| 29 | problem, solv, can, solut, math, one, find, tri, fix, issu, fail, comput, help, understand, simpl |
| 30 | social, news, media, word, can, good, use, bad, one, stori, fake, spread, network, influenc, posit |
| 32 | tech, compani, industri, via, digit, will, futur, busi, world, smart, technolog, startup, new, market, top |
| 36 | read, post, great, thank, blog, share, check, articl, new, stori, piec, write, follow, thought, thread |
| 44 | app, use, googl, new, search, user, power, appl, amazon, product, custom, will, servic, now, site |
| 45 | win, award, competit, congratul, meet, prize, may, congrat, winner, good, won, life, race, final, anoth |
|  |  |
|  | Current issues topics |
| 5 | new, citi, car, map, york, san, area, bike, place, road, south, driver, street, chicago, ride |
| 13 | women, people, white, men, black, gun, american, must, woman, polic, get, man, say, kill, gender |
| 14 | job, tax, cost, pay, year, rate, high, increas, million, market, state, cut, low, billion, incom |
| 17 | trump, vote, presid, elect, american, state, senat, will, lie, hous, democrat, polit, say, aign, law |
| 25 | human, first, space, chang, now, start, robot, earth, expect, right, one, energi, brain, mind, land |
| 28 | chang, can, public, need, will, polici, research, impact, govern, climat, health, make, issu, import, system |
| 33 | health, care, patient, new, medic, studi, risk, doctor, diseas, hospit, drug, help, research, death, cancer |
| 37 | student, school, math, class, educ, colleg, teach, teacher, grad, univers, phd, program, high, today, graduat |
|  |  |
|  | Misc. topics |
| 2 | kid, day, famili, get, today, parent, babi, die, old, said, friend, girl, age, dad, year |
| 19 | food, eat, drink, call, made, order, water, make, hand, ice, coffe, bar, beer, store, yes |
| 24 | game, play, player, team, see, also, season, world, get, first, sport, two, now, start, top |
| 27 | video, watch, love, movi, music, song, amaz, new, star, live, like, world, name, time, art |
|  |  |
|  | Chatter |
| 4 | don, know, want, like, think, peopl, say, thing, feel, just, someth, realli, get, make, can |
| 7 | like, just, say, littl, dog, look, make, can, back, love, good, eye, feel, come, cat |
| 8 | talk, great, today, listen, discuss, podcast, episod, interview, show, watch, miss, present, slide, hear, convers |
| 9 | thank, work, great, amaz, see, much, happi, love, share, mani, team, awesom, excit, hope, everyon |
| 10 | one, love, photo, see, like, light, red, black, wear, got, can, right, size, fun, blue |
| 11 | just, actual, thing, think, lot, one, didn, well, now, realli, right, love, got, use, wasn |
| 16 | work, thing, peopl, think, make, can, much, time, get, way, good, still, one, like, lot |
| 26 | ever, one, time, best, never, first, seen, life, come, thing, alway, real, world, everi, person |
| 31 | year, last, day, week, time, ago, one, two, month, first, old, everi, next, past, sinc |
| 35 | time, day, get, just, back, now, can, start, next, work, take, hour, everi, run, leav |
| 38 | will, take, long, may, hope, come, think, time, well, way, good, back, term, soon, end |
| 40 | ask, question, answer, know, one, right, person, can, mani, get, yes, name, peopl, say, call |
| 41 | can, know, don, let, get, just, need, want, help, tell, now, make, peopl, one, see |
| 42 | like, look, forward, good, see, realli, well, work, great, cool, sound, thank, feel, someth, seem |
| 43 | think, make, peopl, also, like, point, doesn, use, just, mani, mean, agre, case, differ, isn |
